# *ERCC1* mRNA levels and survival of advanced gastric cancer patients treated with a modified FOLFOX regimen

**DOI:** 10.1038/sj.bjc.6604317

**Published:** 2008-03-25

**Authors:** J Wei, Z Zou, X Qian, Y Ding, L Xie, J J Sanchez, Y Zhao, J Feng, Y Ling, Y Liu, L Yu, R Rosell, B Liu

**Affiliations:** 1 Department of Oncology, Drum Tower Hospital, Clinical Cancer Institute of Nanjing University, Medical School of Nanjing University, Nanjing 210008, China; 2 Medical Oncology Service, Catalan Institute of Oncology, Hospital Germans Trias i Pujol, Badalona 08916, Spain; 3 Department of Preventive Medicine and Public Health, Autonomous University of Madrid, Madrid 28029, Spain; 4 Department of Epidemiology and Biostatistics and Data Analysis Center, School of Public Health, Nanjing Medical University, Nanjing 210019, China; 5 Department of Oncology, Jiangsu Cancer Hospital, Nanjing 210009, China; 6 Department of Oncology, Changzhou Cancer Hospital, Suzhou University, Changzhou 213001, China

**Keywords:** *ERCC1*, *TS*, advanced gastric cancer, FOLFOX

## Abstract

Molecular markers involved in DNA repair can help to predict survival in gastric cancer patients treated with 5-FU plus platinum chemotherapy. Excision repair cross-complementing 1 (*ERCC1*) and thymidylate synthase (*TS*) mRNA expression levels were assessed in advanced gastric cancer tumour samples using real-time quantitative PCR in 76 patients treated with a modified FOLFOX (biweekly oxaliplatin plus 5-FU and folinic acid) regimen. Median survival time in patients with low *ERCC1* levels was significantly longer than in those with high levels (15.8 *vs* 6.2 months; *P*<0.0001). Patients with high *TS* levels had longer survival than those with low levels (12.2 *vs* 10.1 months; *P*=0.01). Forty-eight patients with low *ERCC1* and high *TS* levels had a median survival of 16.1 months (*P*<0.0001). The hazard ratio for patients with high *ERCC1* expression was 9.4 (*P*<0.0001). In patients with high mRNA levels of *ERCC1*, alternative chemotherapy regimens should be considered.

Gastric cancer currently ranks the third among most common cancers and will remain a significant cancer burden in China during the next decade. In 2005, there were approximately 400 000 new cases and 300 000 deaths from gastric cancer (Yang, 2006). There is currently no established standard regimen for advanced gastric cancer; however, biweekly oxaliplatin plus 5-fluorouracil (5-FU) and folinic acid (FOLFOX) was well tolerated and yielded median survival times of 9.6–11.4 months in five phase II studies (Louvet *et al*, 2002; Al-Batran *et al*, 2004; Chao *et al*, 2004; De Vita *et al*, 2005; Lordick *et al*, 2005). Considerable published evidence suggests that interindividual variation in response to 5-FU results from variations in the thymidylate synthase (*TS*) mRNA expression levels of the tumour (Longley *et al*, 2003). A sensitive quantitative polymerase chain reaction (QPCR) permits the detection of mRNA in small quantities that could not be detected by conventional methods used to detect proteins. *TS* mRNA levels in frozen endoscopic biopsies were inversely related to response and survival in 65 gastric cancer patients receiving neoadjuvant 5-FU plus cisplatin; median survival was 43 months for those with low *TS* levels and 6 months for those with high levels (Lenz *et al*, 1996). However, some tumours expressing relatively high levels of *TS* mRNA were surprisingly sensitive to 5-FU plus cisplatin (Lenz *et al*, 1996; Metzger *et al*, 1998). Intriguingly, it has recently been reported that gastric cancer cells that were rendered resistant to oxaliplatin exhibited significantly decreased *TS* levels, which resulted in enhanced susceptibility to 5-FU cytotoxicity. In addition, host cell reactivation assay revealed enhanced DNA repair of oxaliplatin damage in the resistant cells (Chen *et al*, 2007).

Cytotoxicity from cisplatin and other platinum-containing drugs, such as oxaliplatin, results from the formation of platinum DNA adducts (Schellens *et al*, 1996; van de Vaart *et al*, 2000). One determinant of the level of platinum DNA adducts in the tissue of patients treated with platinum-containing drugs is the rate of DNA repair. Individuals vary considerably in their capacity to remove DNA adducts, and tumour cell lines demonstrating *in vitro* resistance to cisplatin rapidly clear DNA adducts from the cells (Bosken *et al*, 2002). Nucleotide excision repair (NER) is the major pathway for repairing platinum-induced DNA damage. A series of proteins act to recognise base damage, unwind DNA, remove the damaged single-stranded fragment and synthesise a correct strand in its place (de Laat *et al*, 1999; Rosell *et al*, 2007). Excision repair cross-complementing 1 (*ERCC1*) is a key enzyme in the NER pathway. *ERCC1* and *TS* mRNA levels were examined in 38 of the 65 gastric cancer patients (Lenz *et al*, 1996), for whom sufficient cDNA was available. Median survival for patients with low *ERCC1* mRNA levels was not reached, while it was 5.4 months for those with high levels (*P*=0.034). The differences in median survival according to *TS* mRNA levels were not significant (Metzger *et al*, 1998).

To further clarify the prognostic value of *TS* and *ERCC1* mRNA expression, we examined by QPCR 76 formalin-fixed paraffin-embedded tumour samples from FOLFOX-treated advanced gastric cancer patients and correlated the results with survival.

## PATIENTS AND METHODS

### Patients

Patients with histologically proven locally advanced or metastatic gastric cancer and Eastern Cooperative Oncology Group (ECOG) performance status (PS) ⩽2 were included in the study. All patients received a modified FOLFOX regimen chemotherapy after resection of primary tumours, as follows: oxaliplatin 85 mg m^−2^ on day 1, plus folinic acid 200 mg m^−2^ as a 2 h infusion followed by bolus 5-FU 300 mg m^−2^ and a 22 h infusion of 5-FU 600 mg m^−2^ on days 1 and 2, every two weeks. Survival was calculated from the date of diagnosis to the date of last follow-up or death from any cause. All patients gave their signed informed consent, and the study was approved by the institutional ethics review boards.

### Total RNA extraction from formalin-fixed paraffin-embedded tissue

Seven 7-*μ*m sections were prepared from primary tumour blocks that contained at least 80% tumour cells and directly transferred into a microcentrifuge tube. RNA was extracted according to a previously described method with minor modifications (Specht *et al*, 2001). Briefly, paraffin was removed by extracting three times with 1 ml xylene for 10 min incubation at 50°C followed by rehydration through subsequent washes with 100, 90 and 70% ethanol diluted in RNase-free water. The tissue was collected by centrifugation and resuspended in 200 *μ*l RNA lysis buffer containing 10 mM Tris-HCL, 0.1 mM EDTA, 2% SDS and 500 *μ*g ml^−1^ proteinase K and incubated at 60°C for 16 h. RNA was purified by phenol and chloroform extractions followed by precipitation with isopropanol in the presence of sodium acetate and at −20°C. The RNA pellet was washed in 70% ethanol and resuspended in 20 *μ*l of RNase-free water.

### QPCR assessment of *TS* and *ERCC1* expression

cDNA was generated with a blend of random hexamers and oligo-dT 1 : 1 (ABgene, Surrey, UK) and the target cDNA sequences were amplified by quantitative PCR in a fluorescent temperature cycler (Mx 3000P Real Time PCR System, Stratagene). Briefly, total RNA 1 *μ*g was used for each RT reaction. The 20 *μ*l PCR reaction mixture contained 1 × primers and probe mixture (Applied Biosystems, Foster city, CA, USA. Assay IDs: Hs00157415_m1 (*ERCC1*); Hs00426591_m1 (*TS*); Hs99999903_m1 (*β-actin*)), 1 × Absolute QPCR Mix (ABgene). The PCR conditions were 50°C for 2 min, 95°C for 15 min, followed by 45 cycles at 95°C for 15 s and 60°C for 1 min. Relative gene expression quantifications were calculated according to the comparative *C*_t_ method using *β-actin* as an endogenous control and commercial human total RNA (BD Clontech, CA, USA) as calibrators. Final results were determined by the formula 2^−ΔΔ*C*t^ (Livak and Schmittgen, 2001) and were analysed with the Stratagene analysis software.

### Statistical analysis

The Mann–Whitney *U*-test was used to test significant association between gene expression levels and dichotomous variables. The Spearman's rho method was used to correlate expression levels of *TS* and *ERCC1*. The maximal *χ*^2^ method of Miller and Siegmund (Miller and Siegmund, 1982) and Halpern (Halpern, 1982) was adapted to determine which cutoff value best dichotomised patients into low- and high-expression *TS* and *ERCC1* subgroups; the Tree method (LeBlanc and Crowley, 1992) was then applied to optimise these cutoff values. The final cutoff values were confirmed by recursive partitioning and amalgamation using S-Plus software, version 6.1 (Statistical Sciences, Seattle, WA, USA). Survival curves were obtained by the Kaplan–Meier method. Comparisons were made with the log-rank test and 1000 bootstrap-like simulations were performed to get the corrected *P*-values of the log-rank test. A univariate Cox model with overall survival as the dependent variable was constructed and categorised with gene expression levels as independent variables, and the genes that were significant in the univariate analysis were included in a multivariate Cox proportional hazards model for survival. All statistical analyses were carried out at a 5% level of significance and with a power of 80%, using the Statistical Package for the Social Sciences, version 13 (SPSS Inc, Chicago, IL, USA).

## RESULTS

### Patient characteristics

A total of 76 gastric adenocarcinoma patients were included in the study. The median age was 57; 56 patients were male and the majority of patients had PS 0–1. Twenty-one patients (27.6%) had stage IIIA, 16 (21.1%) had stage IIIB and 39 (51.3%) had stage IV disease at the time of diagnosis. Patient characteristics are summarised in Table 1.

### Gene expression levels

*ERCC1* and *TS* levels were detected in all tumours, with median gene expression relative to housekeeping *β*-*actin* for *ERCC1* of 0.039 (range 0.001–5.23, 95% confidence interval (CI): 0.26–0.83) and for *TS* of 8.83 (range 0.72–78.79, 95% CI: 8.03–12.34). Using a cutoff value of 0.47, 61 patients (80.3%) had low *ERCC1* expression levels and 15 (19.7%) patients had high levels. Using a cutoff value of 6.06, 55 (72.4%) patients had low *TS* expression levels and 21 (27.6%) had high levels. There was no significant association between *ERCC1* and *TS* levels (*ρ*=–0.1, *P*=0.39). *ERCC1* mRNA expression levels were lower in patients with PS 0–1 (median value: 0.017) than in those with PS 2 (median value: 0.099) (*P*=0.03). Distal gastric cancer was present in 62.3% of patients with low *ERCC1* levels and proximal gastric cancer was present in 53.3% of patients with high *ERCC1* levels (*P*=0.02). No significant association was detected between *ERCC1* or *TS* mRNA expression and other clinical parameters.

### Survival

Median survival time for all patients was 11.5 months (95% CI: 8.4–14.6 months). A significant association was observed between survival and PS (*P*=0.02), tumour stage (*P*<0.001) and number of sites involved (*P*=0.004). No other association between clinical characteristics and survival was found (Table 1).

**Figure 1:**
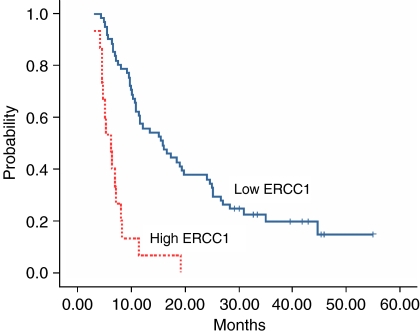
Kaplan–Meier estimates of overall survival by *ERCC1* mRNA expression levels (*N*=76; low *ERCC1*: 61; high *ERCC1*: 15).

A significant association was also observed between survival and gene expression levels. Median survival for patients with low *ERCC1* expression levels was 15.8 months (95% CI: 10.2–21.5 months) compared with 6.2 months (95% CI: 4.6–7.9 months) for those with high levels (*P*<0.0001) (Table 2, Figure 1). Median survival for patients with high *TS* expression levels was 12.2 months (95% CI: 3.7–16.4 months) compared to 10.1 months (95% CI: 5.4–18.9 months) for those with high levels (*P*=0.01) (Table 2).

**Figure 2:**
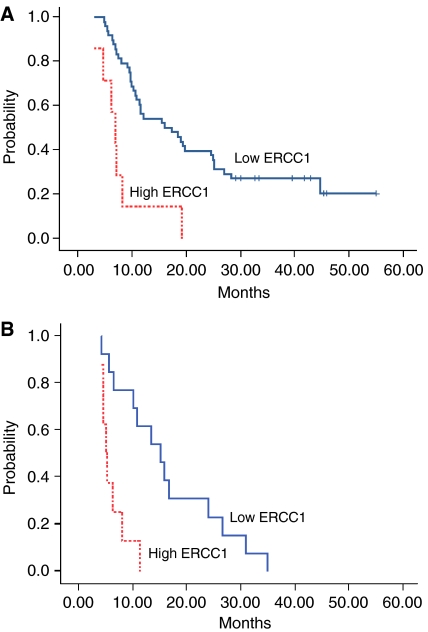
Kaplan–Meier estimates of overall survival according to *ERCC1* mRNA expression in patients with high *TS* mRNA expression (**A**) (*N*=55; high *TS* and low *ERCC1*: 48; high *TS* and high *ERCC1*: 7) and in patients with low *TS* expression (**B**) (*N*=21; low *TS* and low *ERCC1*: 13; low *TS* and high *ERCC1*: 8).

Among the 55 patients with high *TS* expression levels, 48 patients with low *ERCC1* levels had a median survival of 16.1 months (95%CI: 7.8–24.4) compared to 6.9 months (95% CI: 5.2–8.6) for the seven patients with high *ERCC1* levels (*P*<0.0001) (Figure 2A). Among the 21 patients with low *TS* expression levels, 13 patients with low *ERCC1* levels had a median survival of 15.1 months (95% CI: 9.2–21.1) compared to 5 months (95% CI: 4–6.1) for the 8 patients with high *ERCC1* levels (*P*=0.001) (Figure 2B).

The multivariate analysis identified *ERCC1* mRNA expression levels (hazard ratio (HR), 9.4; *P*<0.0001) and number of sites involved (HR, 1.9; *P*=0.03) as independent markers for survival (Table 3).

## DISCUSSION

In this first QPCR analysis of *ERCC1* and *TS* mRNA expression in formalin-fixed paraffin-embedded tumour tissue in advanced gastric cancer, we have found that *ERCC1* – but not *TS* – mRNA expression is associated with survival in patients receiving a modified FOLFOX regimen. These findings are along the same lines as the seminal study by Metzger *et al* (1998) where *ERCC1* and *TS* mRNA levels were quantified in frozen tumour tissue from gastric cancer patients receiving 5-FU plus cisplatin. Since the time of writing the present report, another study in paraffin-embedded tumour tissue has confirmed the role of *ERCC1* mRNA expression in a heterogeneous cohort of gastric cancer patients – 69 treated with S-1, 23 with 5-FU and 43 with cisplatin plus either irinotecan or S-1. In the multivariate analysis of all 140 patients, *ERCC1* emerged as an independent prognostic marker of survival (HR, 2.4; *P*<0.001); however, *TS* mRNA expression was not significant (HR, 0.3) (Matsubara *et al*, 2008). A study assessing *ERCC1* and *TS* by immunostaining in 64 advanced gastric cancer patients treated with FOLFOX also showed significant differences according to *ERCC1* expression. Median survival for patients with negative *ERCC1* was 12.8 months, while it was 8.4 months for those with positive *ERCC1* (*P*=0.03). The multivariate analysis revealed a significant impact of ERCC1 expression on survival (HR, 1.9; *P*=0.04) (Kwon *et al*, 2007). However, TS expression was not found to be related to response or survival (Kwon *et al*, 2007), which concurred with the findings of a previous study (Choi *et al*, 2001). However, in the present study, using QPCR, the differences are much more striking (15.8 *vs* 6.2 months; *P*<0.0001), with a HR of 9.4. The determination of relative gene expression through QPCR is currently considered to be a more sensitive and more quantitative methodology than immunostaining. Several studies have found no correlation between mRNA and protein levels of *ERCC1* (Britten *et al*, 2000; Niedernhofer *et al*, 2007). When *ERCC1* expression at the mRNA and protein levels was assessed by northern and western blotting, respectively, in a panel of cervical carcinoma cell lines, there was a significant correlation between *ERCC1* mRNA – but not *ERCC1* protein – levels and cisplatin resistance (Britten *et al*, 2000). In stage IV, non-small-cell lung cancer patients treated with cisplatin-based chemotherapy, *ERCC1* protein expression did not predict response (Wachters *et al*, 2005), whereas *ERCC1* mRNA expression was significantly associated with response (Cobo *et al*, 2007). Previous studies have tested *ERCC1* mRNA expression in paraffin-embedded tumour samples in advanced colorectal cancer patients treated with 5-FU plus oxaliplatin (Shirota *et al*, 2001) and in non-small-cell lung cancer patients treated with gemcitabine plus cisplatin (Lord *et al*, 2002); both studies found a significant association between *ERCC1* expression and survival. Cisplatin resistance is multifactorial, also involving the copper transporter ATP7A (Chen *et al*, 2007) and a 45-gene expression signature was a predictor of cisplatin sensitivity with 83% accuracy (Hsu *et al*, 2007). Interestingly, this gene signature includes *ERCC1* and other DNA repair genes.

It is not clear, either in the Metzger study (Metzger *et al*, 1998) or in the present one, whether *ERCC1* is a prognostic or a predictive marker. Interestingly, in the present study, ERCC1 mRNA levels were lower in patients with good PS, which has also been found in non-small-cell lung cancer patients (Cobo *et al*, 2007).

The role of *TS* in predicting chemosensitivity remains controversial (Choi *et al*, 2001; Kwon *et al*, 2007; Matsubara *et al*, 2008). Although a meta-analysis reported that colorectal cancer patients with higher *TS* levels had poorer overall survival than those with lower levels (Popat *et al*, 2004), this predictive value was not confirmed in a prospective study (Smorenburg *et al*, 2006). In the present study, patients with higher *TS* levels had longer survival than those with lower levels, especially in patients with lower *ERCC1* levels. Low levels of *TS* have been observed in oxaliplatin-resistant gastric cancer cell lines (Chen *et al*, 2007), providing a plausible potential explanation of why a meaningful number of patients with low *ERCC1* and high *TS* levels had the longest survival in the present study (Figure 2).

Irinotecan and taxane-based regimens have been used in the treatment of advanced gastric cancer patients, with survival times similar to those attained with FOLFOX (Moehler *et al*, 2005; Oh *et al*, 2007; Takayama *et al*, 2007). However, the short survival attained in FOLFOX-treated patients with positive protein *ERCC1* (HR, 1.91) (Kwon *et al*, 2007) or overexpression of *ERCC1* mRNA (HR, 9.4) suggests that irinotecan or taxane-based regimens could be the better alternative for these patients. A randomised customised trial is warranted in this setting.

## Figures and Tables

**Table 1:** Clinical factors associated with overall survival

	**Patients**			
**Characteristics**	**No.**	**Percentage**	**MST** **(Months)**	***P* Log-rank test**
*Age, years (median: 57; range: 30–80)*				
<57	36	47.4	15.7	0.943
⩾57	40	52.6	10.7	
				
*Sex*				
Male	56	73.7	11.5	0.780
Female	20	26.3	11.7	
				
*ECOG PS*				
0–1	62	81.6	11.8	0.020
2	14	18.4	8.1	
				
*Initial staging*				
III	37	48.7	19.2	< 0.001
IV	39	51.3	9.6	
				
*Grading*				
G2	20	26.3	10.7	0.972
G3	56	73.7	11.8	
				
*Site of tumour*				
Proximal stomach	28	36.8	9.8	0.45
Distal stomach	42	55.3	12.2	
Whole stomach	6	7.9	11.3	
				
*No.of sites involved* a				
0–1	49	64.5	16.9	0.004
⩾2	27	35.5	7.6	

Abbreviation: MST: median survival time.

a Sites involved: lymph nodes, liver, pancreas, peritoneum, lung, pleura.

**Table 2:** *ERCC1* and *TS* mRNA expression and survival in advanced gastric cancer patients

**Factors**	**No. of patients**	**MST (Months) (95% CI)**	***P* a**
*ERCC1 mRNA*			
Low ⩽0.47	61	15.8 (10.2–21.5)	<0.0001
High >0.47	15	6.2 (4.6–7.9)	
			
*TS mRNA*			
Low ⩽6.06	21	10.1 (5.4–18.9)	0.01
High >6.06	55	12.2 (3.7–16.4)	

a Adjusted *P*-value based on log-rank statistics after 1000 bootstrap simulations.

**Table 3:** Multivariate analysis of factors associated with overall survival

**Factors**	**No. of patients**	**Hazard Ratio (95% CI)**	***P***
*ERCC1 mRNA*			
Low ⩽0.47	61	1 (ref.)	
High >0.47	15	9.4 (4.1–21.7)	<0.0001
			
*Initial staging*			
III	37	1 (ref.)	
IV	39	1.6 (0.9–2.9)	0.08
			
*ECOG PS*			
0–1	62	1 (ref.)	
2	14	1.8 (0.9–3.3)	0.07
			
*No.of sites involved*			
0–1	49	1 (ref.)	
⩾2	27	1.9 (1.1–3.3)	0.03
			
*Site of tumour*			
Whole stomach	6	1 (ref.)	
Proximal stomach	28	4.4 (1.3–14.5)	0.02
Distal stomach	42	3.8 (1.1–16.6)	0.04
